# *Cordyceps militaris* as a Bio Functional Food Source: Pharmacological Potential, Anti-Inflammatory Actions and Related Molecular Mechanisms

**DOI:** 10.3390/microorganisms10020405

**Published:** 2022-02-10

**Authors:** Abdul-Rehman Phull, Madiha Ahmed, Hye-Jin Park

**Affiliations:** 1 Department of Food Science and Biotechnology, College of BioNano Technology, Gachon University, Seongnam 13120, Korea; ab.rehman111@yahoo.com; 2 Shifa College of Pharmaceutical Sciences, Shifa Tameer-e-Millat University, Islamabad 44000, Pakistan; pharmacist_madiha@hotmail.com

**Keywords:** *Cordyceps militaris*, inflammation, polysaccharides, pharmacokinetics, COX-2, matrix metalloproteinases

## Abstract

*Cordyceps militaris* (*C. militaris*) is a medicinal mushroom possessing a variety of biofunctionalities. It has several biologically important components such as polysaccharides and others. The diverse pharmacological potential of *C. militaris* has generated interest in reviewing the current scientific literature, with a particular focus on prevention and associated molecular mechanisms in inflammatory diseases. Due to rising global demand, research on *C. militaris* has continued to increase in recent years. *C. militaris* has shown the potential for inhibiting inflammation-related events, both in in vivo and in vitro experiments. Inflammation is a multifaceted biological process that contributes to the development and severity of diseases, including cancer, colitis, and allergies. These functions make *C. militaris* a suitable functional food for inhibiting inflammatory responses such as the regulation of proinflammatory cytokines. Therefore, on the basis of existing information, the current study provides insights towards the understanding of anti-inflammatory activity-related mechanisms. This article presents a foundation for clinical use, and analyzes the roadmap for future studies concerning the medical use of *C. militaris* and its constituents in the next generation of anti-inflammatory drugs.

## 1. Introduction

Inflammation is a complex process that occurs as a result of various chemical or physical substances in the body, including pathogens, trauma, toxic substances and others [1]. Type of inflammatory stimulus, and the effectiveness of the response, determine the nature of inflammation as acute or chronic. Augmented synthesis of inflammatory mediators has been associated with chronic ailments such as cancer, arthritis, asthma, viral diseases, atherosclerosis, and others [2]. As a result, slowing down inflammatory events has become crucial, and non-steroid anti-inflammatory medicines (NSAIDs) are commonly utilized for this purpose. NSAIDs have a number of side effects, including renal failure, bronchospasm, gastrointestinal problems, water retention, and hypersensitivity responses [3]. 

As a result, substantial attention has recently been given to the development of natural anti-inflammatory products with greater safety and minimal side effects. Natural products have been in continuous use since prehistoric times for the alleviation of diseases and overall wellbeing. The modern system of medicine was initiated through the isolation of pharmacologically active morphine, which served as the basis for the construction of a therapeutic empire composed of compounds isolated or derived from natural sources [4]. Previously the shifting of paradigm from isolation from natural sources towards synthesis or combinatorial chemistry urged scientists to focus on the large-scale synthesis of drug substances. Due to considerable efforts and reduced productivity in the synthetic process, recent decades have witnessed the regaining of involvement of natural products in drug development in conjunction with new technological approaches such as high-throughput selection [4,5]. Traditional acceptance, cost effectiveness and unique chemical diversity of metabolites have led to exploitation of natural resources for the identification of various pharmacological leads and scaffolds, serving the benefit of humanity [6]. 

**Figure 1:**
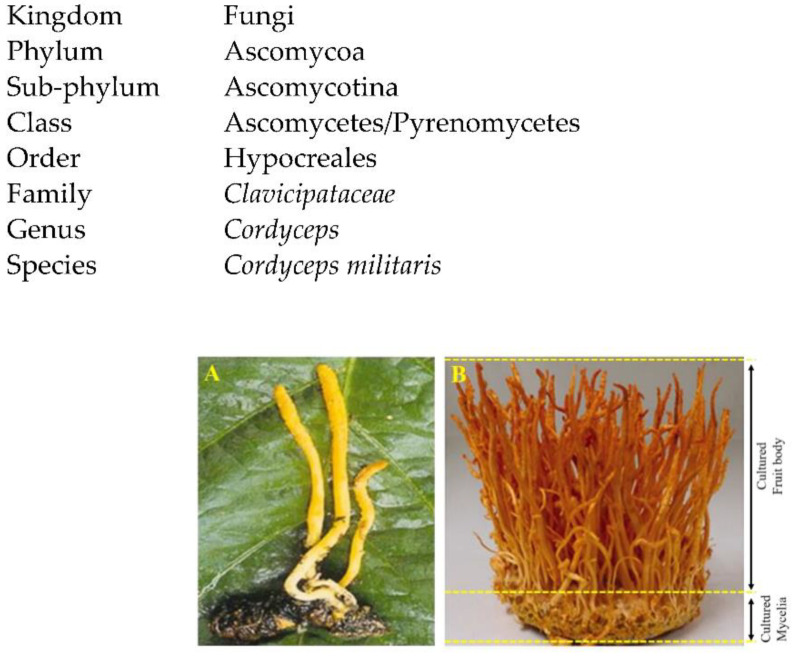
Natural (**A**) and cultivated (**B**) *C. militaris*.

Medicinal mushrooms have remained an important component of human culture. The genus *Cordyceps* is one of the largest genera in the family *Clavicipitaceae*, containing over 750 species, and being extremely diverse in terms of the number of species, their morphology and acclimatization on varied hosts [7,8]. These diverse species are mostly distributed in Asian countries (such as Korea, Japan, Nepal, China), and other parts of the world in humid temperate and tropical habitats. Occurrence of a variety of species in different environmental conditions throughout the world indicates its global distribution [9,10]. Specialized and coordinated mechanisms are involved in the association between the *Cordyceps* species and related hosts. They maintain their life cycles according to the characteristics of the hosts for the growth and survival, after evading their immune systems and subsequent production of defensive secondary metabolites by the hosts, which can be considered as promising sources of new drugs [9]. Based upon this property, these species have gained profound importance as a source of natural products having diverse biological activities [10]. Recently, *Cordyceps* species growing in the wild have been surpassed by artificially cultured specimens due to the scarcity and high price associated with collection and processing [11]. *C. militaris* is an ethnomedicinal fungi having importance in traditional Chinese medicines and extensively used as a crude drug and a functional food in Asia [12]. *C. militaris* is the second most popular, investigated species in its genus. Several pharmacological activities of this species have been documented, including blood glucose control, hypolipidemic, anti-tumor, anti-microbial, anti-viral, antiprotozoal, anti-inflammatory, neuroprotective, anti-oxidant and immuno-protective activities [10,12]. Therefore, the *C. militaris* may be considered an important candidate for the treatment of different ailments. Mycological data of *C. militaris* is given below [12]. Natural and cultured *C. militaris* are shown in Figure 1. 

In today’s world, the concept of “prevention is better than cure” is prevalent, resulting in the establishment of safety and therapeutic profiles of food items. Functional foods can be categorized as foods marketed under the labels of healthy food and food sources having physiological properties besides their nutritional uses. Additionally, natural materials that can be used daily for regulating or affecting the body system upon intake can also be called functional foods [13,14]. According to the European Consensus, the most accurate definition is that “a food can be considered as functional beyond adequate nutritional effects, if it reasonably improves target functions in the body in a way that contributes to improving health and minimizing disease risk”. Functional foods are natural substances; however, different constituents can be incorporated or removed from them through biotechnological procedures [15] to improve health, or help avert diseases in individual or defined groups based upon gender and age [16,17]. The existence or deliberate addition of certain factors such as fibers, flavonoids, polyphenols, anthocyanins, minerals, fatty acids and carotenoids, among others, enhances the nutritional value of the dietary components. Vitamins, minerals and other nutritional components were formerly incorporated in the manufacturing of functional foods. Prebiotics and probiotics, which have a tendency to target metabolic syndrome and other ailments [18,19], along with high blood pressure, cellular damage, oxidation [18,20], are well documented functional foods. These have unique immune-modulatory and immune-boasting potential, and they can enhance immunity through symbiotic association with humans [12]. Apart from treatment functionalities, *C. militaris* might be a good candidate for consideration as functional food due to the presence of metabolites of therapeutic or protective capacities. 

An extensive number of bioactive constituents have been identified from this species including cordycepin, ergosterol, carotenoids, mannitol, proteins, essential amino acids, volatile oils, carotenoids, minerals, vitamins, nucleosides, and sterols, as well as several types of carbohydrates such as mono, oligo, and polysaccharides, which shows its pharmacological and palliative significance [12]. The industrial sector in China is involved in large-scale fermentation and commercial cultivation of its stromata, which is attributed to a decline in the wild population, and satisfies the increasing demand for medicinal, edible and nutritional purpose [21]. Further, the bioactive compounds produced in fermented culture is a major reason for its industrial production [21,22]. Currently, at least 36 health foods prepared from this medicinal fungus have been approved in China. Moreover, health food products of *C. militaris* mycelia in form of powder (Z20030034) and capsules (Z20030035) have gained the status of commercially approved moieties with claims of having beneficial effects on kidneys and lungs, and being effective against cough, asthma, phlegm, cold limbs, fatigue, dizziness, tinnitus, and other ailments. Furthermore, since the Chinese Ministry of Health recognized it as a Novel Food in 2009, it has been widely consumed as a regular food item [23].

The objective of the present study is to provide a comprehensive update on the pharmacological effects of *C. militaris* in terms of inflammation prevention, as well as the mechanisms of action at the molecular levels that may contribute to its anti-inflammatory properties.

### 1.1. Chemical Constituents 

#### 1.1.1. Proteins and Peptides 

Fresh raw material is preferred over processed or dried parts for the isolation of the maximum content of bioactive peptides and proteins from medicinal mushrooms [24]. An important peptide, cordymin, isolated from *C. militaris* is about 10.9 kDa in size and its N-terminal sequence makes it unique among other peptides. Cordymin was observed to inhibit many fungal species, such as *Rhizoctonia solani*, *Mycosphaerella arachidicola* and others. It is also reported to decrease the proliferation of MCF-7 breast cancer cells and levels of HIV-1 reverse transcriptase [25]. Additionally, it has preventive effects against osteopenia by increasing bone mineral content and density in diabetic rats [26]. Another lectin, named CML, has been isolated from *C. militaris* by gel filtration chromatography, and is approximately a 31 kDa peptide. It is distinctive from the lectins identified from several mushroom species including *Polyporus squamosus*, *Laetiporus sulphureus*, and others in terms of differences in secondary structure, containing 29% β-turns, 12% β-sheets, 27% α-helix, and 32% random-coil structures. CML has been observed to exert hemagglutinating activity in experimental animals by specifically combining sialoglycoproteins. This compound has also shown to have mitogenic activity on mouse splenocytes [27]. Similarly, another report discussed the isolation of *C. militaris* protein (CMP). It has also exhibited antiproliferative effects on human breast cancer MCF-7 cells (IC_50_ 9.3 μM) and bladder cancer 5637 cells (IC_50_ 8.1 μM). It also possesses antifungal activity against *Fusarium oxysporum* at a minimum concentration of 1.6 μM [28]. Three protein-polysaccharide polymer complexes (CPSP-F1, CPSP-F2, and CPSP-F3) have been isolated form by *C. militaris* using an ultrasound-extraction and column chromatography technique. Among these compounds, CPSP-F1 and CPSP-F2 exhibited inhibitory IC_50_ as 32.2 ± 0.2 mg/mL and 5.3 ± 0.0 mg/mL in an acetylcholinesterase inhibitory assay, suggesting its ability to be used against Alzheimer’s disease [29]. A fibrinolytic enzyme has also been produced by *C. militaris* using a submerged fermentation technique. The specific enzyme was purified from culture supernatant by hydrophobic interaction, ion exchange and gel filtration chromatographic strategies. It prevented clot formation by degrading the α, β and γ chains of fibrinogen, and also activated plasminogen into plasmin, thus proving to be a good anticoagulant agent [30]. Another isolated novel protein is “*C. militaris* immune-regulatory protein” (CMIP), which showed anti-metastatic activity on a 4T1 breast cancer lung metastasis, numbers of tumor nodules in the lung, and improved survival rate in experimental animals [31]. 

#### 1.1.2. Polysaccharides 

Polysaccharides in fungal species are among the most prevalent and significant biologically active constituents extracted from fruiting bodies, mycelium and fermentation broth. These substances show diverse physicochemical properties and have been the target of developing quality control of mushrooms, especially *C. militaris*, containing functional foods [32]. In this species, polysaccharides have been divided into two types i.e., intracellular polysaccharides and extracellular polysaccharides, based on their formation or origin in fungal cells. The polysaccharides of *C. militaris* are reported to exhibit a range of biofunctions, such as immunomodulatory, antioxidant, antitumor and anti-inflammatory effects. A novel polysaccharide (PCLM) obtained from the culture broth of *C. militaris* enhanced the immune-stimulatory activity of RAW264.7 macrophages by modulating the release of toxic molecules (nitric oxide (NO) and super oxide dismutase (SOD)) and cytokine tumor necrosis factor (TNF)-α, as well as inducing phagocytosis [33]. Another report suggested that *Cordyceps* polysaccharides have the tendency to overcome CY-induced immunosuppression, and significantly enhanced the function of spleen lymphocytes and macrophages [34]. A previous study reported CMP-W1 and CMP-S1 (two polysaccharides) showed significant enhancement of proliferation of spleen cells from an animal model [35]. In another finding, the functional polysaccharides CMP40 and CMP50 enhanced lymphocyte proliferation, interleukin (IL)-4 concentrations and antibody titers in the serum [32]. The polysaccharides obtained from *C. militaris* also possess strong antioxidant activities. Three polysaccharides extracted from *C. militaris* exhibited strong anti-radical activity. The W-CBP50II polysaccharide was observed to have more activity against hydroxyl, superoxide and 1,1-diphenyl-2-picrylhyrazyl (DPPH)-radical scavenging activity [36]. Other novel polysaccharides such as CBP-1, P70-1 extracted and purified from the fruiting body of cultured *C. militaris*, exhibited strong hydroxyl radical scavenging activity with an IC_50_ value of 0.638 mg/mL and 0.548 mg/mL, respectively [32,37]. The exopolysaccharides of mutant *C. militaris* SU5-08 enhanced adaptive immune responses. Further, these polysaccharides have shown anticancer activity against a variety of cell lines. The polysaccharide CMP-I significantly inhibited the proliferation of HepG2, HeLa, K562 and HT29 cells [38]. Another report showed the dose-dependent inhibitory potential of *C. militaris* derived polysaccharides against BGC-823, MCF-7, and SMMC-7721 cells [39]. Another study reported the antiproliferative potential of a polysaccharide from *C. militarism* against adenocarcinomic human alveolar basal epithelial cells (A549) cells, with an IC_50_ of 39.08 μg/mL [38]. Additionally, the report confirm the inhibitory effects of *C. militaris* polysaccharides on colon 205, NCI-H460, PC-3 cell lines [40,41]. These components also inhibited tumor in animal model [42]. 

Yuko et al., reported a *C. militaris*-derived acidic polysaccharide (APS) containing arabinose (Ara), galacturonic acid, D-Gal, Rha, and Xyl with an MW of 5.76 × 10^5^ Da. It showed beneficial health improving properties on influenza A virus infection via modulation of the macrophages related to immune functions [43]. Another study investigated the beneficial effects of a *C. militaris*-derived polysaccharide on maturation of dendritic cells (DC), suggesting that it might be used in cancer immunotherapy [44]. The protective effects of a *C. militaris*-derived polysaccharide on hydrogen peroxide-stimulated apoptosis in HL-7702 cells was investigated previously. The tested polysaccharide significantly reduced hydrogen peroxide-stimulated apoptosis, mitochondrial dysfunction, production of reactive oxygen species (ROS), decreased intracellular adenosine triphosphate levels, and augmented secretion of cytochrome C. [45]. The polysaccharides from cultured *C. militaris* have demonstrated anti-aging potential by preventing mitochondrial injury. It was observed that the tested sample inhibited Fe^2^(+)-L Cysteine activated swelling and mitochondrial damage in a dose-dependent manner [46]. In other studies, polysaccharides of *C. militaris* were found to boost immunological effectiveness against Newcastle disease vaccination in animals. The study suggested that the polysaccharides might be contenders for a novel form of immunological adjuvant [47]. Various studies on the isolation of polysaccharides from this species have been documented, such as CPS-1 polysaccharide from *C. militaris* composed of d-glucose (D-Glc), xylose (Xyl), rhamnose (Rha), d-mannose (D-Man), and d-galactose (D-Gal), with a molecular weight of 2.3 × 10^4^ Da, which was extracted and reported as an immune-modulating agent [48]. Other polysaccharides such as cordlan [44], cysinocan consisting of D-Gal, D-Man, D-Glc, with an MW of 8.2 × 10^4^ Da [49], two hetero-polysaccharides (CMP-W1; 3.66 × 10^5^ Da and CMP-S1; 4.6 × 10^5^ Da) consisting of D-Glc, D-Gal and D-Man, have been reported as immunomodulatory agents [35]. The polysaccharide CMPB90-1 with an MW of 5.8 × 10^3^ Da was studied as a tumor eradication agent through immune regulatory activity [50]. Polysaccharide structure, including monosaccharide content and glycosidic linkages, is intimately associated with their immunomodulatory action. TLR2 is most likely to identify polysaccharides containing galactose and glucose, which regulate immunomodulatory functions [51,52]. Polysaccharides with α-d- and β-d-glucosidic bonds have been found to have a more positive impact on NO generation in macrophages. Therefore, it can be inferred that immunomodulatory potential of the CMPB90-1 polysaccharide may be attributed toward its monosaccharide like galactose and glucose. A previous study demonstrated polysaccharide mediated induction of M1 polarization, along with TLR2 as the membrane receptor of MC-2 on macrophages. These polysaccharides were structurally identical to CMPB90-1 [51,53]. The structure of CMPB90-1 regulates its activity on the phenotype of macrophages 

#### 1.1.3. Nucleosides

**Figure 2:**
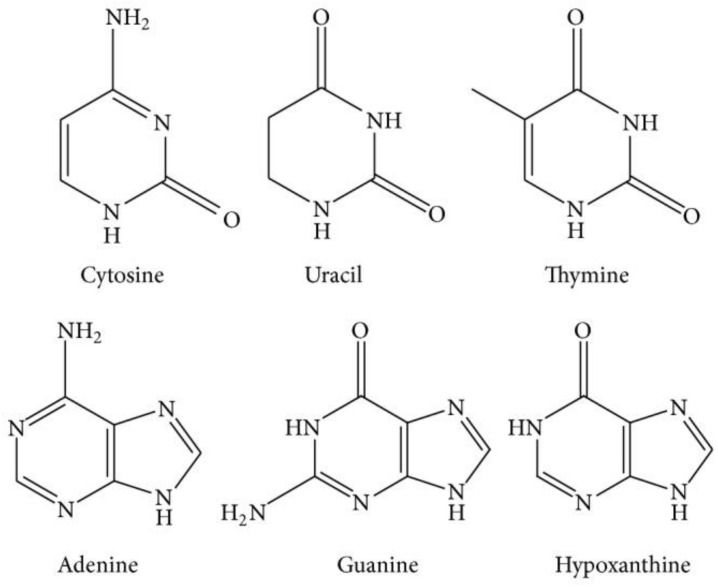
Chemical structure of six nucleosides.

*C. militaris* contains nucleosides and nitrogenous bases (adenosine, guanosine, cytidine, uridine, adenine and uracil) as its main components, among which, adenosine significantly suppressed the release of neurotransmitters in the central nervous system and could be used to treat chronic heart failure, and also tonically inhibits the release of excitatory neurotransmitters [54]. This nucleoside is reported to be a neuronal modulator and to inhibit neuronal action, including regulation of the sleep cycle, regulation of seizure susceptibility, and locomotor effects. Pharmacologically it can be used as an analgesic agent, and also mediates the effects of ethanol and chronic drug use. Additionally, it is important as a neuroprotection agent [55]. Adenosine receptor subtypes in blood arteries have been identified and characterized, and when triggered have a considerable influence on peripheral circulation [56]. Another nucleoside, cytidine, has shown antidepressant-like effects in a forced swim test with rat models [57]. Moreover, intraperitoneal and oral administration of guanosine can inhibit α-dendrotoxin- and quinolinic acid-induced seizures in experimental animals [58]. The chemical structures of various nucleosides are presented in Figure 2. 

**Figure 3:**
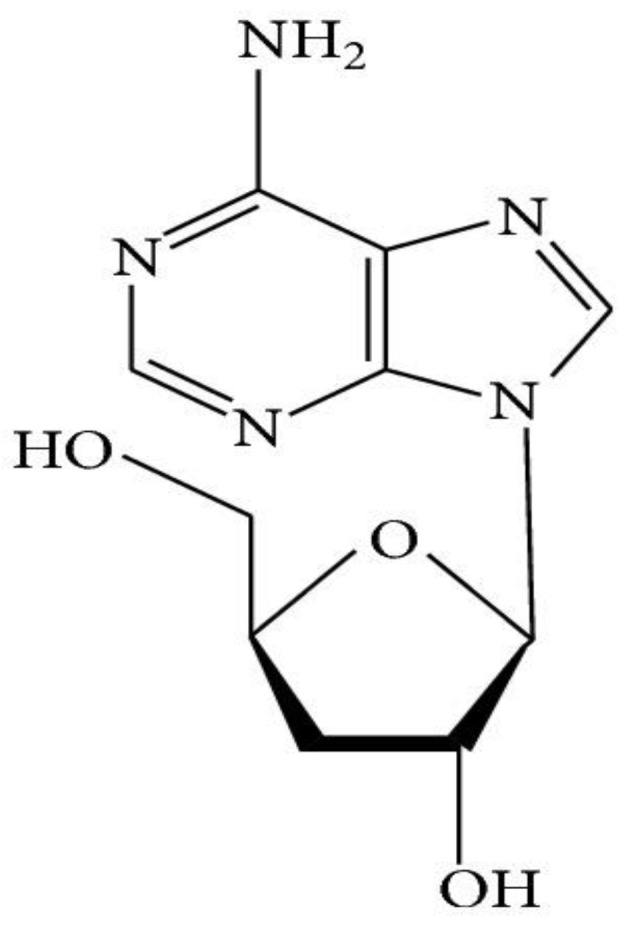
Chemical structure of cordycepin.

Cordycepin, also known as 3′-deoxyadenosine, is a bioactive metabolite that was initially found in the fermented broth of *C. militaris* [59]. It is a nucleoside analogue that has been proved to possess an array of biological activities including antibacterial, antifungal, antitumor, antileukemia, and antiviral activities, as well as an immunoregulatory effect [60,61]. Different mechanisms of action have been identified, including inhibition of purine synthesis, RNA chain termination and interference in mTOR signal transduction [62]. Cordycepin has various pharmacological functions, one being antidiabetic activity. This compound has proved to be effective in reducing the blood glucose levels as well as blood glucose tolerance levels of alloxan-induced diabetic mice [63]. The mechanism of its antidiabetic activity is not yet fully understood. However, different reports investigated that cordycepin can suppress the production of proinflammatory cytokines (such as IL-1β, IL-6, TNF-α and NO) in LPS stimulated macrophages, leading to downregulation of type 2 diabetes-regulating genes (11β-hydroxysteroid dehydrogenase type 1 and peroxisome proliferated activated receptor-λ) [64]. It inactivates nuclear factor kappa B (NF-κB)-regulated inflammatory responses and corresponding reduced expression of diabetes-regulating genes [65,66]. Cordycepin has also been observed to exert anti-hyperlipidemic activity due to its structural resemblance to adenosine (activating adenosine mono phosphate kinase-AMPK), which can lead to inhibition of Acetyl CoA carboxylase and result in reduction of fatty acid synthesis [67]. It can also reduce the levels and accumulation rate of low-density lipoprotein cholesterol, triglycerides and total cholesterol in an effective manner [68]. Pertaining to its lipid lowering activities, this compound can be recognized as a prominent bioactive agent for cardiovascular illnesses treatment. It also enhances the cytokine release of resting peripheral blood mononuclear cells (PBMCs), and proliferation and transcription factors in PBMCs are amplified in a leukemia cells (THP-1), suggesting an immunomodulatory function [69]. Pure bioactive constituents isolated from *C. militaris* have demonstrated immunomodulatory effects, lowering the generation of anti-ds-DNA and boosting the survival rate of lupus mice [20]. An antiosteoporotic effect of cordycepin has been observed in osteopenic rat models, in which loss of bone was retrieved in the test animals. The reduced activity of alkaline phosphatase enzymes and tartrate-resistant acid phosphatase was also found in a variety of experiments models. Cordycepin consumption can induce the levels of osteocalcin (prominent marker of bone development), reduce oxidative stress and decrease the C-terminal cross-linked telopeptide of type I collagen content (bone resorption marker) in ovariectomized experimental animals [70]. The effects of cordycepin against malarial parasites in mice have been detected, in which the nucleic acids and protein synthesis of the parasites ceased [71]. Cordycepin is also a good anti-inflammatory, antihyperuricemic, and antioxidant agent, and considered to have the ability to treat respiratory disorders, kidney malfunctioning, arthritis, cancer and fertility issues [20]. In addition, the main component, cordycepin itself, has demonstrated antitumor, anticancer, insecticidal and antimicrobial activities [8]. The structure of cordycepin is shown in Figure 3. 

#### 1.1.4. Phenolic Compounds 

Medicinal mushrooms have a significant number of phenolic compounds, specifically polyphenols and flavonoids. These compounds have vital roles as antioxidants with various mechanisms including metal chelation, free radical scavenging, inhibition of LDL oxidation and enzyme modulation activities [72]. Yu et al., detected the presence of polyphenolic (60.2 μg/mL) and flavonoid (0.598 μg/mL) content in cultured *C. militaris* extracts [73]. Similarly, according to another report, phenols, flavonoids, lycopene, beta carotene and ascorbic acid were detected in methanolic extracts of *C. militaris* by UV visible spectroscopy [74].

#### 1.1.5. Others

Along with the aforementioned constituents, *C. militaris* contains other metabolites that elevates its therapeutic significance. Ergosterol analogues of *C. militaris* are reported to exert their actions as antiviral and antiarrhythmic substances [75]. They have the tendency to suppress activated human mesangial cells as well as alleviate immunoglobulin A nephropathy (Berger’s disease) [76]. In addition, mannitol (sugar alcohol) and trehalose free sugars have been found in *C. militaris*. Mannitol and trehalose free sugars have also been found in *C. militaris*. Mannitol has certain bioactivities such as antitussive, diuretic, free radical inhibition and other bio-functionalities [77]. Certain novel carotenoids have been separated from its fruit bodies and identified as xanthophylls [78]. Pigmented compounds, such as carotene, can be incorporated, and functional foods produced in the food industry acting as improved anticancer functional foods in addition to traditional carotenoids. Moreover, a new cerebroside (glycosphingolipid) cordycerebroside A, along with soyacerebroside I and glucocerebroside, have been isolated from *C. militaris*. These compounds reduce the augmented pro-inflammatory iNOS and suppress cyclooxygenase (COX)-2 in LPS-stimulated monocyte/macrophage-like (RAW264.7) cells [79].

## 2. Pharmacological Actions of *Cordyceps militaris*

**Figure 4:**
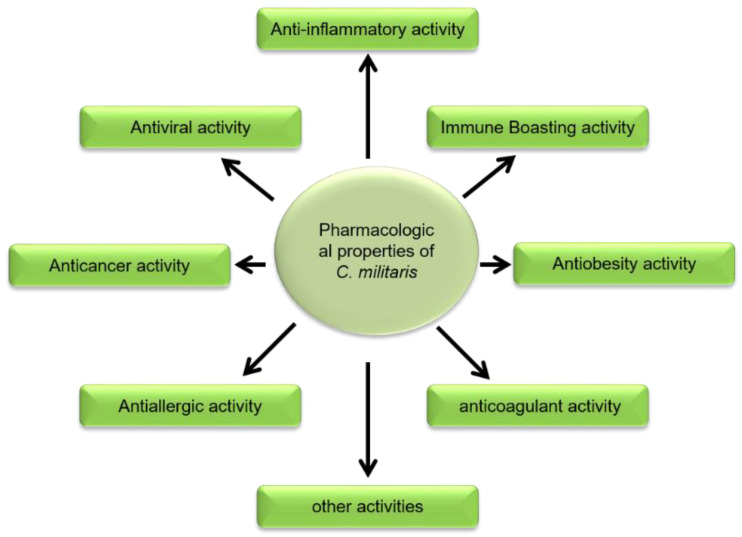
Pharmacological potential of *Cordyceps militaris* and its constituents.

The medicinal mushroom *C. militaris* has been widely consumed in China for medication purpose since ancient times (3000 years). It is used for therapeutic treatment of lung and kidney malfunction, hyperglycemia and hyperlipidemia, respiratory disorders, fatigue, treatment of night sweating, fertility issues, cardiac arrhythmias, and other heart diseases. On a broader scale, *C. militaris* has an array of pharmacological properties, including as inflammation inhibition, and antioxidant, antitumor, antimetastatic, immunomodulatory, hypoglycemic, and steroidogenic activities [12]. Various pharmacological activities are presented in Figure 4 and explained bellow.

### 2.1. Immune Boosting Activity

Several reports have suggested the immune regulation activities of extracts of this medicinal mushroom. The oral administration of aqueous extracts from the *C. militaris* fruiting body at a concentration of 20 mg/kg resulted in induced interferon (IFN) secretion by macrophages via IL-18 [80]. Both fresh and dried *C. militaris* extracts have been observed to have equivalent immunomodulatory effects in clophosphamide (cy)-activated immunosuppressed experimental animals. Quantitative investigation of phytochemicals revealed that levels of cordycepin and adenosine in fresh and dried *C. militaris* were similar, whereas fresh extracts contained more polysaccharides, total polyphenol, and total flavonoids compared to dried ones. Both types of extracts reversed the inhibition of the thymus and spleen index in a dose-dependent manner in a diseased mice model. Additionally, these extracts were able to increase the levels of IL-2 and IFN-γ secretion levels in test animals [81]. An ethyl alcohol extract of *C. militaris* administered to healthy Korean male volunteers enhanced cell-mediated immunity at a dose of 1.5 g/day via an oral route. Researchers observed no side effects after a treatment duration of 4 weeks along, with significant enhancement in the levels of IL-2 and the IFN-γ compared to the placebo group. Further, due to enhanced T cell proliferation and improved natural killer cell activity, this fungus was promoted as a safe immunomodulator to boost cell-mediated immunity [82]. In another study, *C. militaris* fruiting body extracts displayed immune modulation potential and antioxidative activity in healthy Kunming mice. Oral administration of different extracts concentrations i.e., 50, 100, or 200 mg/kg on a daily basis caused a significant increase in thymic and splenic indices. Total white blood cell count, as well as monocytes and lymphocytes, were enhanced, neutrophils were decreased, but eosinophils and basophils did not undergo any changes. Augmented IL and TNF-α levels were reported in the spleen and increased total antioxidant capacity, glutathione peroxidase, and SOD were observed in different organs such as the heart, kidney, and liver. These results suggest an immune boosting response in heathy organisms [46] and indicate the positive impact of *C. militaris* as an immunomodulatory agent. 

### 2.2. Antiviral Potential

*C. militaris* extract has been used to determine its protective effects in influenza A/NWS/33 (H1N1) virus-infected mouse. The virus preventive effects of *C. militaris* extracts were investigated by administering different doses at 30, 100, or 300 mg/kg per day for seven days to H1N1-infected animals. The results inferred that the protective effect of the extracts could be attributed to suppressed TNF-α level along with increased IL-12, natural killer (NK) cells in experimental mice models [83]. Viral hepatitis occurs commonly worldwide, and when untreated causes hepatocellular carcinomas and cirrhosis. *C. militaris* showed moderate anti-HCV potential in conjunction with standard antivirals (IFN-α or ribavirin) in a cell-based HCV RNA replication assay system used to investigate antiviral activity [84].

### 2.3. Anticoagulant Activity

Fibrin is formed by fibrinogen by the activity of thrombin. The accumulation of fibrin in blood vessels can lead to clot formation resulting in thrombosis and cardiovascular diseases. A number of reports have confirmed the presence of fibrinolytic enzymes in *C. militaris* with anti-coagulant or thrombolytic activities. Cui et al., successfully purified a novel fibrinolytic enzyme from culture broth of the species and named it *C. militaris* fibrinolytic enzyme (CMase) [85]. Similarly, Liu et al. purified a fibrinoyltic enzyme, which had the ability to hydrolyze fibrin or fibrinogen by cleaving the α-chains more efficiently than β- and γ-chains, revealing its plasmin like nature. It was able to degrade thrombin, indicating its benefits as an anticoagulant and antithrombotic protein [86]. The same authors reported the biochemical characterization of another fibrinolytic protease from *C. militaris* [30]. Hence, *C. militaris* can be suggested as good source of novel thrombolytic agents, although work related to the anticoagulant activities of crude extracts is scarce. 

### 2.4. Anticancer Activity

*C. militaris* is one of the important medicinal species and well documented for its anticancer effects. *C. militaris* ethanolic extract was orally administered to a xenograft in mice bearing murine T cell lymphoma (RMA) cell-derived cancers, which resulted in significant anticancer activity by the suppressing the size and mass of cancer. Furthermore, reduced proliferation of RMA cells and C6 glioma cells, downregulation of phosphorylation of AKT, p85 and augmented cleaved caspase-3, phosphoglycogen synthase kinase 3β (p-GSK3β) were reported. The extract significantly increased the proapoptotic cell population and reduced viability compared to control cells. The finding indicates the anticancerous activity of *C. militaris* occurred by regulating of p85/AKT- or GSK3β-related caspase 3-dependent apoptosis [87]. Similarly, methanolic extracts showed good cytotoxic activity via the MTT assay against Hep-2 cancer cell lines with an IC_50_ value of 20 μg/mL [74]. In another study, the effect of fluoride was monitored in the culture medium of *C. militaris*, and positive effects were observed on the synthesis of secondary bioactive metabolites and growth of fruiting bodies, which eventually caused reduced proliferation and apoptosis in a human osteosarcoma (U2OS) cell line [88]. Another study discussed the decreased apoptotic activity of aqueous extract of *C. militaris* (AECM) on MDA-MB-231 cells. It showed significant induction of mitochondrial dysfunction and loss of mitochondrial membrane permeability by modulating Bcl2/Bax proteins, and also caspase activation [89]. Another report showed the tumor inhibitory effects of an ethanolic extract of *C. militaris* in xenograft Balb/c nude mice transfected with human colorectal carcinoma RKO cells. The oral administration of test extracts led to delayed growth of RKO cell-derived tumors. It also stimulated cell cycle arrest in G2/M phase (66.33% at 300 μg/mL) and enhanced early apoptosis (18.07% at 300 μg/mL). Western blot analysis indicated an increase in the expression levels of p53, cleaved caspase 9, cleaved caspase-3, cleaved PARP, and Bim, Bak, and Bad proteins [90]. A mechanistic based study conducted by Chou et al., revealed that anticancerous effects of *C. militaris* on leukemia cell lines might be attributed to activation of AKT and p38 mitogen activated protein kinase (MAPK), during the course of apoptosis induction, suggesting the possible use of its extracts against leukemia by activating the p38 MAPK pathway [91]. Another mechanism of the apoptosis of lung carcinoma by *C. militaris* extracts is related to downregulation of TCTN3 expression, which affected the hedgehog signaling cascade and contributed to the serial activation of caspases. Additionally, the extract negatively modulated GLI1 transcriptional activity by inhibiting SMO/PTCH1 molecules, subsequently regulating the intrinsic apoptotic signaling cascade [92]. All these findings support the possible use of *C. militaris* extracts as anticancer agents in future studies.

### 2.5. Anti-Obesity Activity

*C. militaris* extracts possess lipid lowering activities. A novel extract of mulberry leaves fermented with *C. militaris* was exploited to detect its effect on lipid metabolism. Administration of an extract in a high fat diet fed to (HFD)-activated obese C57BL/6 mice for 12 weeks showed significantly decreased concentrations of triglyceride, glucose, total cholesterol and low-density lipoprotein, and induced production of levels of high-density lipoproteins were observed. The amount of abdominal fat and the size of adipocytes were reduced compared to control groups. Moreover, the sample reduced the Fas cell surface death receptor for lipogenesis and inhibited adipocyte protein 2 and peroxisome proliferator-activated receptor-γ mRNA expression [93]. Recently, strawberry extracts fermented with *C. militaris* showed enhanced levels of secondary metabolites as well as different extents of inhibition of adipogenesis in a 3T3-L1 cell line [94,95]. The extract also showed dose-dependent suppressed differentiation of 3T3-L1 preadipocytes into mature adipocytes and did not show any toxic effects on cells. An associated reduction in lipid accumulation, increased levels of adipocyte markers including peroxisome proliferator-activated receptor-γ, adiponectin, and CCAAT/enhancer binding protein-α, as well as continuous expression of monocyte chemoattractant protein (pre adipocytes marker) were observed [96].

### 2.6. Anti-Allergic Activity

Allergic responses are associated with disorders related to the immune system, where intense immune reactions occur in response to various triggers such as foods, chemicals, pollens and particulate matter. Primarily, production of CD4+ specific allergen cells i.e., type 2 helper, Th2 are accompanied by the generation of interleukins (IL-4, IL-5, IL-9, and IL-13) by the effector Th2 cells, which subsequently results in the generation of IgE i.e., allergen related immunoglobins from B cells. IgE reactions with allergen cytokines generate the allergic reaction. Therefore, suppression of both allergen cytokines and IgE are effects of therapeutic agents for allergies. Aqueous extracts of *C. militaris* have demonstrated asthma-preventing potential in ovalbumin (OVA)-activated experimental animals at a dose of 4 g/kg/day. Results revealed a decreased concentration of serum immunoglobulin E (IgE) as well as fewer infiltrating cells in the airways of mice treated with test extracts, although efficiency was less compared to montelukast and steroids, which are standard drugs for the treatment of asthma [97]. In another study, *C. militaris* (ethyl acetate extract) inhibited allergic reactions in a concentration-dependent manner in basophilic leukemia (RBL-2H3) cells. Extracts inhibited antigen-activated degranulation in RBL-2H3 cells with an IC_50_ value of 28.5 μg/mL. The extract prevented antigen-induced passive cutaneous anaphylaxis in experimental animals in a concentration-dependent manner [98]. In another study, an extract of *C. militaris* cultured on germinated soybean extract showed inhibitory potential in 2,4-dinitro-1-fluorobenzene (DNFB)-activated contact dermatitis mice models at a concentration of 300 mg/kg. It not only led to reduced ear swelling but also reduced infiltration of T, CD4 and CD8 cells in the ear tissues of the mice [99]. A study was performed to determine the molecular mechanisms of allergy prevention. An ethyl alcohol extract prepared from silkworm pupa-cultivated *C. militaris* fruiting bodies in immunogen triggered RBL-2H3 mast cells inhibited the release of β-hexosaminidase (a degranulation marker) and mRNA levels of TNF-α, as well as IL-4. Western blotting results revealed the inhibition of the Syk/phosphatidylinositol 3-kinases (PI3K)/MEKK4/JNK/c-Jun signaling cascade associated with the expression of various allergic cytokines in stimulated RBL-2H3 cells. Additionally, inhibited PLCγ evocation, and Erk activation were involved in stimulating the synthesis of lipid mediators and Ca^2+^ mobilization, which favor degranulation in activated RBL-2H3 cells [100]. 

### 2.7. Other

Several studies have documented the antihyperglycemic potential of *C. militaris*. Oral administration of aqueous and ethanolic extracts of *C. militaris* to diabetic Sprague-Dawley rats caused significant reduction in blood glucose levels. These results were due to increased glucose metabolism and suppression of total cholesterol and triglyceride concentrations [101]. The diabetic preventive potential of different fractions of *C. militaris* in streptozotocin-induced diabetic animals was determined in another study, resulting reduced blood glucose levels in which *C. militaris* extract acted as an insulin sensitizer (enhanced insulin secretion and insulin resistance in type II diabetic rats) [102]. This medicinal fungus has also proved its importance as a fertility enhancer, antimicrobial and antiaging species [103]. 

## 3. Inflammation

**Figure 5:**
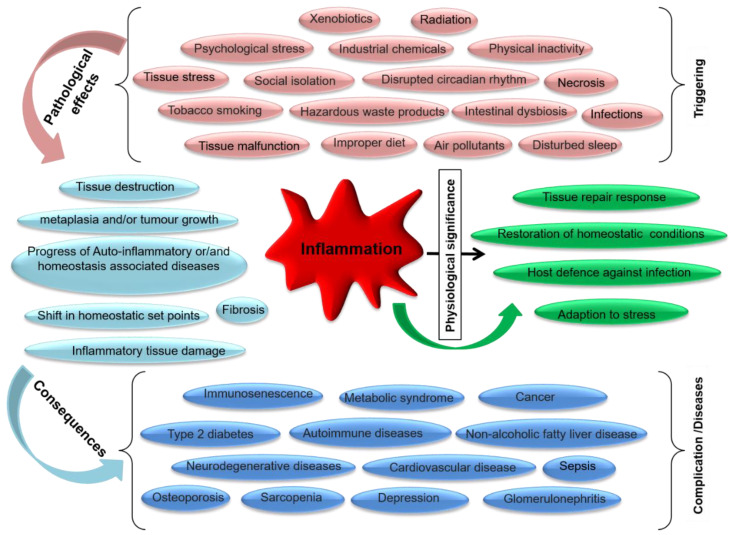
Triggering, pathological consequences, and physiological significance of inflammation.

Inflammation is a key function in biological processes initiated by various stimuli and noxious factors such as irradiation by ultraviolet light, irritants, infections and cell injury. The main features of inflammation are redness, elevated temperature, pain and alteration in physiological functions at infected sites [104,105]. Generally, inflammation is considered to be protective mechanisms against pathogen-induced tissue damage, and it may be acute or chronic. It involves neutrophils, natural killer cells, mast cells, and T and B cells supporting the undesired immune reaction [104]. Prolonged chronic inflammation increases the risk of various inflammation associated disorders such as arthritis, asthma, cancer, and atherosclerosis. A variety of regulatory enzymes such as phospholipase A2 (PLA2), lipoxygenases (LOX), COX, phosphatidylinositol kinase, and tyrosine kinases have a substantial role in inflammation and immune responses. Injured or physiologically altered tissues generate stress, which acts as either an endogenous or exogenous inducer of the inflammatory response [106]. These inducers elicit both mast cells and macrophages residing at inflamed tissues that subsequently promote cellular inflammatory mediators [107]. Elicitation of the inflammatory cascade can proceed through three different paths linked with a variety of pathological events as depicted in Figure 5. Among them, the immune response occurs in infection allied inflammation. Inflammatory mediators such as lipid mediators, chemokines, proteolytic enzymes, complement component fragments, cytokines, vasoactive amines and peptides are characterized on the basis of their biochemistry [108], while IL and TNF-α alter the function of effectors such as cells and tissues during inflammatory responses [109]. Overall, these inflammatory mediators have divergent effects in different cell and tissue types, with a wide range of functions in the inflammatory response, regulating homeostasis and adoption [110].

Stimulators and bacterial endotoxin-triggered immune cells such as leukocytes generate pro-inflammatory cytokines, which participate in elicitation, activation and regulation of cellular adhesion molecules (CAMs). CAMs comprise different groups such as integrins, selectins, and various glycoproteins (immunoglobulins). Tethering, rolling, transmigration to inflammation related areas, and binding of white blood cells and endothelium are all crucial functions. Different studies have demonstrated mechanisms such as migration and mobility white blood cell during inflammation [104]. Inflammatory cells are characterized by augmented inflammation-related chemicals including TNF-α, IL-1, IL-6 and IL-1β, which contribute to inflammation related response such as regulation of chemokines, cytokines and CAMs production. Furthermore, reduced inflammation related reactions have been found against TNF-α and IL-1β specific antibodies [111], showing their involvement in inflammation regulation. 

Arachidonic acid (A.A) has a vital role in inflammation by regulating inflammatory metabolites, and inflammation regulatory enzymes such as LOX, and COX. A.A is one of the prominent contributing factors and biomarkers of inflammation, and its inhibition has been recognized as a molecular target to protect against inflammatory ailments [104]. 

Exogenous factors including microbes and tumor promoters promote inflammatory cells such as macrophages eosinophils, and neutrophils, which are connected with oxidative stress. Various reactive nitrogen species (RNS) and ROS act as fuel for inflammatory processes by prompting the production of proinflammatory cytokines and adhesion related components via NF-κB pathway activation. Augmented inflammatory responses, including activated NF-κB, and induced expression of adhesion molecule have been investigated in oxidative stress activated neutrophils [112]. 

NF-κB is a prominent molecular component that activate and regulates gene transcription [113] of pro-inflammatory chemicals, the A.A cascade, cytokines, chemokines, and inflammatory mediator-induced phosphorylation of I-κB via I-κB kinase and damage to the I-κB complex. It initiates the activation of NF-κB and its related reactions associated with inflammation [114]. Variations in NF-κB pathway activation have been linked with chronic inflammation related ailments such as cancer, inflammatory bowel illness, asthma, atherosclerosis, and rheumatoid arthritis, [115], as well as maintaining different inducible transcription factors in inflammation. Inflammation involves several enzymatic proteins that are related to immune regulation and removal or destruction of various substance through by blood cells [116]. The role of these proteins as inflammatory mediators has long been known. These proteins attach to pathogens after being activated by a recognition protein such as C-reactive protein (CRP), natural IgM, or mannan-binding lectin. 

Inflammatory response results in the production of enzymes involved in degradation of the cellular matrix, such as matrix metalloproteinases (MMPs) [117]. Various biomolecules occur on the surface of leucocytes, such as selectins, which support their entry into tissue spaces by rolling on the endothelial surfaces [104].

Currently, steroidal and nonsteroidal inflammation preventive medications are used to treat acute inflammation and related diseases [118]. However, these medicines are not fully effective against chronic inflammation-related conditions and may have adverse impacts on human health. As a result, it is critical to investigate materials with low adverse effects for the management and treatment of inflammation [119]. Functional foods have attractions in developing novel therapeutics because of their nutritional and pharmacological potential. Furthermore, a lot of attention has been diverted towards natural sources including mushrooms that contain medicinally important biofunctional components that can reduce the severity of inflammatory ailments via different mechanisms such as regulating oxidative stress in the physiological range and controlling pro-inflammatory cytokines. *C. militaris* and its bioactive components are being investigated for biomedical applications, due to their reduced side effects, rich nutritional and bioactive constituents and suppression of inflammation. *C. militaris* and its active constituents have both in vitro and in vivo inflammation preventing potential in a variety of experimental models [120,121,122]. 

## 4. *Cordyceps militaris* and Inflammation

Chronic inflammation causes diseases that are characterized by devastating effects. Many different factors induce inflammation, such as chemicals, physical injury, infectious agents, immunological responses, and metabolic disorders. Medicinal fungi such as *C. militaris*, or their constituents such as cordycepin, are currently being investigated as therapeutic agents against inflammation as they prevent acute and chronic inflammatory responses. The actual mechanisms involved in anti-inflammatory responses include antioxidant activity, transcription factors, matrix metalloproteinases, complement cascade properties, and adhesion molecules such as intercellular adhesion molecule-1 (ICAM-1), selectin, and vascular cell adhesion molecule-1 (VCAM-1). Moreover, *C. militaris*, and its constituents also regulate pro-inflammatory enzyme actions and gene expression of inflammatory genes. Recent reports revealed that Cordycepin, one of the prominent components of *C. militaris*, inhibited inflammation-associated gene expressions of COX-2 and iNOS [123]. Furthermore, ethanol extract of silkworm pupa-cultivated *C. militaris* fruiting bodies inhibit the secretion of histamine, protein kinases [124], and regulate gene transcription associated with inflammation or inflammation associated diseases [125]. Many studies have demonstrated the potential of *C. militaris* in prevention of inflammation. Hence, this study comprehensively reviews the available data on the inhibition potential of *C. militaris* against inflammation along with related molecular mechanisms. The anti-inflammatory mode of action and molecular events linked with suppression of inflammation associated with *C. militaris* in various experimental models are presented in Table 1 and briefly explained below.

### 4.1. Antioxidant Potential 

Oxidative stress arises mainly because of the antioxidant system being unable to remove systematic production of ROS or restore damage produced by the ROS in living systems [112]. RNS are also associated with oxidative stress. Vital biological molecules are susceptible to ROS, and RNS. Their interaction can result in damage of cellular components, causing modifications and altered functioning of bio-molecules such as proteins, DNA and others [112,126]. Changes in these processes result in the activation of inflammatory pathways mediated by inflammatory mediators, which cause damage and altered functioning. Inflammatory cells, such as macrophages and neutrophils, contribute to maintaining oxidative stress. Neutrophils play a vital role in the regulation of inflammatory processes by the generation of superoxides via triggering NADPH oxidase, that leads to a worsening effect in cells [127]. Numerous studies have demonstrated the antioxidant potential of *C. militaris*. Zhang et al. documented the substantial radical scavenging activity of the polysaccharide-iron (III) on DPPH, hydroxyl ABTS, and superoxide species [128]. In another study, the antioxidant capacity of neutral polysaccharide was assessed with assays of reducing power, ABTS radical scavenging activity, oxygen radical absorbance capacity (ORAC-fluorescein), and hydroxyl radical scavenging activity [129]. An ethanol extract of *C. militaris* showed in vitro antioxidant activity against DPPH, superoxide, and hydroxyl radicals and low-density lipoproteins [130]. Extracellular polysaccharide from *C. militaris* effectively regulated key proteins such as HO-1, Nrf2, Kelch-like ECH-associated protein-1 (Keap1), and quinone oxidoreductase 1 (NQO1) in the Nrf2 signaling pathway [131]. He et al. also supported the anti-inflammatory property of an ethanol extract of *C. militaris* via the suppression of H2O2-stimulated cell injury related to ROS overproduction and downregulated mitogen-activated protein kinases in C6 glial cells [132]. The stimulation of redox-sensitive transcription factor Nrf2 averts oxidative stress damage by activating HO-1 [126]. *C. militaris* polysaccharides prominently increased catalase, SOD, glutathione peroxidase levels and total antioxidant capacity, and reduced malondialdehyde (MDA) in experimental animals [34]. Additionally, use of *C. militaris* was a potent therapy for inflammation associated damaging effects in neurological illnesses [132]. Cordycepin suppressed LPS-induced MDA content and inflammatory cytokines (IL-1β, TNF-α) production. It also inhibited LPS-stimulated NF-κB activation, Nrf2 and HO-1 expression [133]. 

### 4.2. Effects on Proinflammatory Enzymes

Various inflammatory mediators including prostaglandins (PGs) i.e., PGE2, 6-keto prostaglandin F1α (6-keto-PGF1α) and other active lipids are found in all human cells. Cyclooxygenases and prostaglandin synthases catalyze a series of events that create PGs from A.A at the site of inflammation [134]. The increased production of iNOS takes place mostly at inflammatory sites, resulting in the augmented synthesis of NO, which ultimately induces synthesis of PGs [119,126]. PLA2 and LOX enzymes are also linked in the regulation of the A.A pathway. Likewise, LOX participates in the derivation of leukotrienes (LT) by the A.A route, which has been connected to inflammatory diseases such as cancer, inflammatory bowel disease, asthma, allergy and other ailments. *C. militaris* ameliorated IL-1β-stimulated COX-2 expression in splenocytes [135]. LPS-induced LPS-stimulated RAW264.7 cells exposed to Cordycepin showed concentration-dependent reduced synthesis of NO and proinflammatory cytokines including IL-6, IL-1β, TNF-α, COX-2 and iNOS, [64]. Another study demonstrated the anti-inflammatory effect of cordycepin attributed to inhibiting NO synthesis, suppressing COX-2 and NF-κB activation, Akt and p38 phosphorylation and iNOS expression [136]. *C. militaris* powder at a dose of 0–3 g per individual suppressed inflammatory cytokines such as EGF, eotaxin, fractalkine, GM-CSF, GRO, G-CSF, IFN-α2, IFN-γ, IL-1α, IL-6, IL-8, IP-10, MCP-1, MIP-1β, MCP-1, MIP-1α, TGF-α, sCD40L, VEGF that were reported in the blood samples from volunteers of both sexes, i.e., male and female [137]. In comparison to the LPS-treated control cells, GRC-ON89A decreased the release of NO. It also suppressed the production of COX-2, iNOS, and TNF-mRNA in LPS-triggered macrophages. In addition, pretreatment with GRC-ON89A suppressed LPS-induced activation of NF-κB and MAPKs (ERK, JNK, and P38) [120].

Leukotrienes are crucial molecules related to inflammatory processes in inflammatory bowel disease cancer, asthma, and rheumatoid arthritis. These molecules are derivatives of the LOX-mediated arachidonic acid pathway [104,126,138,139]. NO is a significant important secondary messenger in the signaling pathway regulating pathophysiological conditions. The advantageous roles of NO in neurological and defensive systems have been widely discussed for many years, and NO has evolved as a mediator of several bio-activities. In addition to these effects, there is a link between increased NO production and inflammation-related disorders. The interaction of NO with metal causes changes in the activity of enzymes such as catalase, which results in H_2_O_2_ accumulation and harmful consequences. Additionally, peroxynitrite is an effective oxidant associated with apoptosis and DNA damage. Oxidation of low-density lipoproteins and suppressing of mitochondrial respiration occurs as an outcome of interaction between superoxide anions and NO [126]. NO tends to promote the synthesis of pro-inflammatory cytokines such as TNF-α and others [104]. *Asterina pectinifera* fermented *C. militaris* extract decreases LPS-triggered expression of inducible NO synthase and inhibited pro-inflammatory cytokines such as TNF-α and IL-6. Furthermore, it ameliorates the LPS-triggered phosphorylation levels of JNK1/2, ERK1/2, and p38 MAPKs in RAW264.7 macrophages [140]. 

### 4.3. Effects on Inflammation-Associated Gene Expression

*C. militaris* and its constituents regulate numerous molecular mechanisms, including PI3K, MAPK pathways, activator of transcription (JAK/STAT) pathways, Janus kinase-Signal Transducer, protein kinase C (PKC), and others. These mechanisms contribute to inflammation and related diseases via regulating inflammatory mediators and are comprehensively discussed in this study. Inflammatory conditions are linked to kinases that affect the production and regulation of transcription factors such as activator protein-1 (AP-1) [141]. 

The MAPK family includes important serine/threonine protein kinases such as c-jun N-terminal kinase (JNK), p38, and extracellular signal-regulated kinase1/2 (Erk1/2) [142]. Inflammatory stimuli initiate these pathways that ultimately control the expression of targeted genes such as TNF-α, IL-1, and COX-2. Cordycepin inhibited LPS-stimulated NF-κB activation and suppressed LPS induced lung wet/dry ratio, MDA content, and inflammatory cytokine (IL-1β, TNF-α) production [133]. *Asterina pectinifera* fermented *C. militaris* extract decreased LPS-induced expression of iNOS and inhibits proinflammatory cytokines such as TNF-α and IL-6. Additionally, it ameliorates the LPS-induced phosphorylation levels of ERK1/2, JNK1/2, and p38 MAPKs [140]. Cordycepin attenuated airway hyper responsiveness, mucus hypersecretion, and OVA-specific immunoglobulin (Ig) E in an experimental model. It blocked severe OVA-induced inflammatory cell recruitment to the lungs such as eosinophils, neutrophils, macrophages, and lymphocytes. It decreased the upregulation of eotaxin, ICAM-1, IL-4, IL-5, and IL-13 in mice. Furthermore, it inhibited p38-MAPK and NF-κB signaling pathway activation in OVA-driven asthmatic mice [143]. An aqueous extract of *C. militaris* significantly reduced the serum levels of MCP-1, ICAM-1, VCAM-1, and NF-κB p65 compared with disease model rats, and total cholesterol, serum creatinine, triglyceride, blood urea nitrogen and urine protein in a cationic bovine serum albumin-induced membranous glomerulonephritis rat model. It also attenuated altered levels of inflammatory factors such as IL, TNF-α and 6-keto-PGF1α, and NF-κB p65. In addition, increased the levels of serum albumin, total protein, MDA, and induced the production of SOD levels and glutathione peroxidase were observed [144]. Cordycepin inhibited IL-1β, PGE2, NO synthesis, along with production of MMP-13, IL-6, iNOS and COX-2 in IL-1β-induced chondrocytes [145]. In another study, cordycepin reduced the production MMP-3, MMP-13, ADAMTS-4, and ADAMTS-5. It also inhibited oxidative stress-associated factors (NO and PGE2) and increased the synthesis of aggrecan and collagen-2 in LPS-activated models [146]. A militarin derivative inhibited the synthesis of NO and PGE2 at the transcriptional level through the inhibition of multiple targets including Syk/NF-kB, IKKe/IRF-3, and p38/AP-1 pathways in LPS-activated RAW264.7 cells and peritoneal macrophages [122]. PI3K has a critical role as a mediator of inflammatory signaling pathways [67]. Stimulation of PI3K leads to AKT activation via a cascade of events that subsequently causes cytokine synthesis [147]. *C. militaris* fermented mulberry (*Morus alba*) leaves exerted anti-inflammatory response through the PI3K/AKT/mTOR signaling pathway in high fat diet-induced obese mice. It reduced mast cell infiltration, inflammatory mediator expression (iNOS and COX-2), and proinflammatory cytokine synthesis (NF-κB, IL-1, -6, and TNF-α) [148]. The anomalous activity of the JAK/STAT pathway in inflammation has been detected in response to various stimuli, resulting in the stimulation of gene transcription related to inflammatory conditions. Various cytokines enhance STAT phosphorylation, which controls gene transcription [104,149]. Similarly, JAK-phosphorylated STATs are implicated in the control of target gene transcription, such as TARC chemokine [150]. 

### 4.4. Effects on Transcription Factors

There are numerous stimuli, such as LPS, RNS, ROS and proinflammatory mediators activating I-κB phosphorylation through I-κB kinase, that break down the I-κB complex and cause NF-κB activation. NF-κB then translocates into the nucleus where it induces the transcription of genes such as IL-1, iNOS, TNF-α, myeloperoxidase lipoxygenases, adhesion molecules, COX-2, and pro-inflammatory cytokines [104,114]. Furthermore, NF-κB activation is linked to a number of degenerative inflammatory diseases such as inflammatory bowel disease, asthma, atherosclerosis, and rheumatoid arthritis [115]. 

Nonsteroidal anti-inflammatory therapeutic agents, including aspirin and sulindac have been shown to inhibit the NF-κB response. The pharmaceutical substance that adverts these pathways can reduce inflammatory processes in living beings. Various reports have shown that *C. militaris* reduces NF-κB-regulated inflammation and related responses in a variety of experiments [40,120,136,148,151]. Various inflammatory genes, such as COX-2 IL-8, IL-6, and TNF-α, are activated by the NF-κB transcription factor, which is susceptible to oxidative stress. Cordycepin attenuates caerulein-stimulated degenerative histological features of pancreatic injury, such as augmented inflammatory cell (neutrophil) infiltration, and reduced edema, acinar cell vacuolization, and serum amylase and lipase levels. It inhibited inflammatory chemicals (IL-6, TNF-α and IL-1β,) by suppressing the activation of NF-κB and NLRP3 inflammasomes in an experimental model [152]. Additionally, *C. militaris* serves as an effective inhibitor of pro-inflammatory cytokines, demonstrating its anti-inflammatory tendency via NF-κB inhibition, and down regulating the iNOS, COX-2, MAPKs and AKT cascades in LPS-mediated inflammations in RAW264.7 macrophages [120]. Repression of RANKL is also linked with events that may be important targets for controlling inflammation-associated condition such as cancer and colitis. *C. militaris* extract demonstrated concentration-dependent suppression of receptor activator of NF-κB ligand (RANKL)-triggered osteoclast differentiation. In addition, cordycepin considerably suppressed RANKL-activated NF-κB and p38 phosphorylation [153].

### 4.5. Effects on Adhesion Molecules

Endothelial adhesion molecules such as vascular cell adhesion molecule (VCAM)-1, P-selectins (platelets), monocyte chemotactic protein (MCP)-1, L-selectin (leukocytes), intracellular adhesion molecule (ICAM)-1, and integrin, play a critical role in the interactions of endothelial and leukocytes during inflammatory processes [104]. These molecules have a variety of functions such as infiltration, adhesion, tethering, and rolling. These are only a few of the critical functions of leukocyte-mediated inflammation. Augmented immunoglobin-G adhesion components, including ICAM-1, VCAM-1 participate in migration, activation of T cells, and leucocyte recruitment [104,154]. In vitro experiments exhibited that diverse inflammatory related molecules, such as IL-1β and TNF-α, cause the synthesis of E-selectin [155], P-selectin [156], and leukocyte adhesion molecules [157] in endothelial cells. This illustrates the implication of proinflammatory cytokines in the recruitment of white blood cells at the site of inflammation. Aqueous extract of *C. militaris* prominently decreased the serum levels of NF-κB p65, VCAM-1, ICAM-1, MCP-1, compared with disease model rats, as well as blood urea nitrogen, serum creatinine, total cholesterol, triglyceride, and urine protein. It also attenuated altered inflammatory chemicals such as IL, TNF-α and 6-keto-PGF1α, and NF-κB p65. Furthermore, increased levels of serum albumin, total protein, MDA, SOD levels, and glutathione peroxidase were observed [144]. *C. militaris* extract showed neuroprotection against focal and permanent ischemic brain damage through anti-inflammatory activities. It suppressed oedema and the infiltration of ED-1-and MPO-positive inflammatory cells into ischemic lesions in an experiment model. It also inhibited chemoattractant (MCP-1)-induced microglial migration [158]. A soya-cerebroside of *C. militaris* attenuates IL-1β-activated monocyte migration and MCP-1 expression and inhibits SP1 levels via upregulation of miR-432 and the phosphorylation of AMPK and AKT [159]. Suppressed inflammatory cytokines including eotaxin, EGF, fractalkine, GRO, GM-CSF, G-CSF, IFN-α2, IFN-γ, IP-10, IL-8, IL-6, IL-1α, MCP-1, MIP-1β, MIP-1α, sCD40L, VEGF, TGF-α were reported in the blood samples of both male and female volunteers [137]. Extravasion of neutrophil was effectively inhibited in the peritoneal inflammatory model. Increased selectin receptors are linked to inflammatory diseases including pancreatitis. Various noninfectious inflammatory ailments such as acute pancreatitis are characterized by high mortality and morbidity, and accompanied by tissue necrosis and severe inflammation. Cordycepin inhibited a variety of proinflammatory cytokines, including TNF-α, IL-6, and IL-1β via downregulating NLRP3 inflammasomes and NF-κB inhibition in experimental model [152].

### 4.6. Effects on Matrix Metalloproteinase

The enzyme inhibitory potential of *C. militaris* against a variety of enzymes including COX, matrix metalloproteases (MMPs) and others has been well reported. Inhibition of these enzymes confines tissue injuries in various diseases, reduces inflammation and mitigates metastasis. MMPs are a group of zinc-containing calcium-dependent proteases (endopeptidases) involved in the breakdown and remodeling of extracellular matrix components [160]. Hormones, growth factors, and cytokines all have a role in regulating MMP expression. 

Tissue inhibitors of metalloproteinases (TIMPs) control MMPs in the physiological range. Increased or decreased TIMP or MMPs can trigger different diseases or increase their severity including inflammation-related ailments [161]. 

Proinflammatory cytokines such as IL-1, IL-17, TNF-α and others induced the production of MMPs in bovine primary chondrocytes, chondrosarcomal cells (SW1353), and human primary chondrocytes [104,162]. Cordycepin inhibited IL-1β-stimulated MMP-1 and MMP-3 expression in rheumatoid arthritis synovial fibroblasts in a concentration-dependent manner. It also suppressed IL-1β-stimulated p38/JNK and AP-1 activation [163]. *C. militaris*-derived soya-cerebroside inhibits IL-1β-induced MMP-1 synthesis by regulating MEK, ERK, FAK, and AP-1 signaling cascades [159].

**Figure 6:**
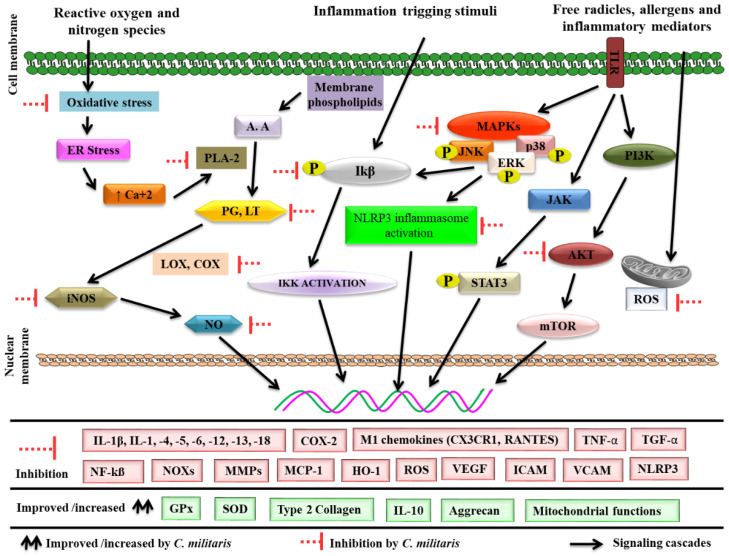
*C. militaris* and its constituents associated anti-inflammatory molecular mechanisms. Arachidonic acid: A.A; Cyclooxygenase 2: COX-2; Heme oxygenase-1: HO-1; Interleukin-1β: (IL)-1β, -4, -5, -6, -10, -12, -13, -18; Intracellular adhesion molecule: ICAM; Inducible nitric oxide synthase: iNOS; Leukotrienes LT; Lipoxygenases: LOX; Monocyte chemotactic protein-1: MCP-1; Matrix metalloproteinases: MMPs; Nuclear factor-κB: NF-κB; phospholipase A2: PLA-2; Glutathione peroxidase: GPx; Prostaglandin: PG; Superoxide dismutase: SOD; Transforming growth factor-α: TGF-α; Tumor necrosis factor alpha: TNF-α; Vascular endothelial growth factor: VEGF; Vascular cell adhesion molecule: VCAM. Upward double arrow shows the improved/increased content or functionality. Single black arrow indicates signaling cascades, while the red symbol specifies the inhibition of inflammation associated signals and biomolecules.

*C. militaris* associated anti-inflammatory molecular mechanisms are depicted in Figure 6.

**Table 1:** Biofunctional components, concentration, experimental/disease model and anti-inflammatory actions of *Cordyceps militaris*.

Bioactive Component	Dose/Disease Model	Study Type/Experimental Model	Results/Mechanism	References
Cordycepin	2.5–10 mg per kg of rat/Parkinson’s disease	In vivo/ Male Sprague-Dawley rats	Reduced neuro-inflammation, dynamin-related protein 1 (Drp1), IL-1β, IL-18 and tyrosine hydroxylase. Amplified NLRP3 inflammasome activation, ATP production, AMP-activated protein kinase and mitochondrial functions	[164]
Cordycepin	0.0005–0.008 nM/L	In vitro/ PC12 rat pheochromocytoma cell line	Improved mitochondrial functioning by increased ATP content, maintaining membrane potential, inhibiting fission protein 1(Fis1) and mitochondrial ROS levels.	[164]
Cordycepin	0–40 µg per mL/ TNF-α-induced inhibition of osteogenic differentiation in ADMSCs	In vitro/ ADMSCs	Restoration of cell proliferation and osteogenic differentiation by regulating Runx2 and Osx mRNA expressions, and NF-κB signaling via inhibition of IκBα phosphorylation.	[165]
Cordycepin	0–40 µg per mL/LPS-stimulated RAW264.7 cells	In vitro/ RAW264.7 cells	Reduced proinflammatory chemicals such as IL-1β, IL-6, TNF-α, iNOS, COX-2 and NO synthesis	[64]
*C. militaris* extract (WIB801C)	20, 50, 100 mg per kg of rat/Focal cerebral ischemia	In vivo/ Male Sprague-Dawley rat	Neuroprotection, inhibited MCP-1-induced microglial migration, oedema and the infiltration of ED-1-and MPO-positive inflammatory cells.	[158]
*Asterina pectinifera* fermented *C. militaris* extract (FACM)	0–40 µg per mL/LPS-induced RAW264.7 macrophages	In vitro/ RAW264.7 macrophages	Amelioration of LPS-stimulated phosphorylation levels of MAPKs (p38, JNK1/2, and ERK1/2), NO synthase expression, IL-6 and TNF-α.	[140]
Cordycepin	10, 20, 400 mg per kg of rat/ Acute lung injury, asthma.	In vivo/ Male BALB/c mice	Inhibited OVA-specific immunoglobulin (Ig) E, mucus hypersecretion, eotaxin, IL-4, -5, -13 and ICAM-1, NF-kB activation and p38-MAPK signaling cascades, recruitment of inflammatory cells in an experimental model.	[143]
Militarin Derivatives	0–100 µM/ LPS-treated RAW264.7	In vitro/ RAW264.7 cells, peritoneal macrophages	Inhibited NO production and PGE2 by downregulating p38/AP-1, IKKe/IRF-3, and Syk/NF-kB pathways	[122]
Militarin Derivatives	5–20 mg per kg in DSS-induced colitis, 5–30 mg per kg in gastritis model and ear oedema model	In vivo/ male C57BL/6 and ICR mice	Anti-inflammatory effects by reducing gastric damage (gastritis), inhibited colon size and up-regulated phospho-p38 (colitis), and inhibited ear oedema.	[122]
Cordycepin and adenosine	0, 1, 10 and 100 µg per mL/ LPS induced inflammatory response	In vitro/ Murine macrophage	Inhibition of inflammation by reducing expression of M1 chemokines (CX3CR1, RANTES) and cytokines (IL-1β, TNF-α).	[166]
Extract of *C. militaris* grown on soybean	5–20 mg per kg of mice/ DSS-induced colitis	In vivo/ C57BL/6 mice	Inhibited TNF-𝛼, iNOS, MMP-3, MMP-9 mRNA Expressions in colonic tissue of a colitis model.	[167]
Extract of *C. militaris* grown on soybean	10 and 100 µg per mL/ LPS-induce RAW264.7 Cells.	In vitro/ RAW264.7 cells	Suppressed TNF-𝛼 and iNOS in a cell model	[167]
Extract (Mulberry leaves fermented with *C. Militaris*)	High fat diet-induce -obese mice	In vivo/ C57BL/6N male mice	Inhibited mast cell infiltration, COX-2, iNOS, IL-6, -1β, TNF-α, NF-κB. Anti-inflammatory response via the PI3K/AKT/mTOR signaling pathway.	[148]
Cordycepin	0, 10, 50 or 100 µM/ Nucleus pulposus cell and intervertebral disc organ culture inflammatory models	In vitro/ rats	Increased type-II collagen, aggrecan synthesis. Inhibited PGE2, NO, and matrix damaging enzymes (MMP-3, -13; ADAMTS-4, and -5).	[146]
*C. militaris* extract, fractions, ergosterol	0.1, 1, 10 and 100 µg per mL/ LPS-stimulated BV2 microglia cells	In vitro/ BV2 microglia cells	Significantly reduction in LPS induced nitric oxide.	[168]
*C. militaris*-fermented product extract	0.603–1.809 g per kg per day/ liver fibrosis BALB/c mice	In vivo/ Male BALB/c mice	Suppressed proinflammatory cytokines, such as TNF-α, IL-6, and NF-κB.	[151]
Cordycepin, *C. militaris* butanol extract	0–30 µg of Cordycepin per mL/ or 0–75 µg of extract per mL/ LPS-triggered RAW264.7 cells	In vitro/ RAW264.7 cells	Anti-inflammatory effect by inhibiting NO synthesis, NF-κB activation, iNOS, COX-2 expressions and phosphorylation of p38 and Akt.	[136]
Ergosterol palmitate; palmitic acid; ergosterol; ergosterol peroxide; 3,4-*O*-isopropylidene-d-mannitol; Cordycepin; d-mannitol; d-glucose	LPS/IFN-α stimulated murine peritoneal macrophage cells	In vitro/ macrophage cells	Suppressed synthesis of cytokines including IL-12 and TNF-α and NO production	[40]
Soya-cerebroside, *C. militaris* extract	0, 1, 5, and 10 µM/ IL-1β-induced monocytes	In vitro/ Monocyte	Reduced monocytes migration and MCP-1 expressions. Downregulated SP1 expression by activating miR-432 and inducing phosphorylation of AKT and AMPK.	[159]
Soya-cerebroside, *C. militaris* extract	3 and 10 mg per kg per day/ IL-1β-induced inflammatory rat model	In vivo/ Severe combined immunodeficiency	Inhibited edema and cartilage damage. Induction in CD68 and MCP-1 (a marker for monocyte/macrophages) positive cells,	[159]
Soya-cerebroside	0, 1, 5, and 10 µM/ Osteoarthritis synovial fibroblasts (OASFs)	In vitro/ humans	Decreased monocyte migration, activated AKT and AMPK signaling pathways, MCP-1 and microRNA (miR)-432 expression in OASFs.	[159]
*C. militaris* extract	1, 10, 100 and 1000 µg per mL/ In LPS-stimulated RAW264.7 and antigen-induced RBL-2H3 cells	In vitro/ RAW264.7 and RBL-2H3 cells	Inhibited nitrite production, iNOS, and TNF-α.	[169]
*C. militaris* extract	500 mg per kg of animal per day/ DSS induced acute colitis	In vivo/ BALB/c mice	Alleviated the severity of the disease in a colitis mouse model by decreasing mRNA expression of TNF- α and iNOS.	[169]
GRC, GRC-ON89A	250, 500 µg per mL/ LPS-induced Macrophages	In vitro/ RAW264.7 cells	Reduced NO production, iNOS, COX-2, and TNF-α mRNA expression, and that of MAPKs (ERK, JNK, and P38), NF-κB.	[120]
GRC, GRC-ON89A	25 mg per kg of animal/ DNFB induced allergic contact dermatitis	In vivo/ BALB/c, C57BL/6N mice models	Decreased inflammatory response such as ear swelling in an experimental model	[120]
Cordycepin	12.5, 25, 50, 100 µg per mL/ Cholecystokinin-stimulated pancreatic acinar cancer cell	In vitro/ pancreatic acinar cancer cell	Anti-inflammatory effect by down regulating NLRP3 inflammasome activation and NF-κB via AMPK.	[152]
Cordycepin	100 mg per kg of animal/ Caerulein induced acute pancreatitis	In vivo/ Male ICR mice	Augmented neutrophil infiltration and reduced edema, acinar cell vacuolization, serum amylase, lipase levels. Inhibited TNF-α, IL-1β, IL-6 by suppressing the activation of NLRP3 inflammasome and NF-κB.	[152]
*C. militaris* aqueous extract	1 and 2 g per kg of animal/ Cationic bovine serum albumin-induced membranous glomerulonephritis rat model	In vivo/ Wistar male rats	Amplification of total protein, serum albumin, MDA, SOD, and glutathione peroxidase. Attenuated IL-1, TNF-α, 6-keto-PGF1α, NF-κB p65. Reduced serum levels of VCAM-1, ICAM-1, and MCP-1 and urine protein serum creatinine, triglyceride, blood urea nitrogen and total cholesterol.	[144]
Extract of fruiting bodies *C. militaris*	500 µg per mL/ LPS-induced inflammatory response in macrophages	In vitro/ RAW264.7 Macrophages	Reduced Synthesis of IL-6, NO, and TNF-α.	[170]
Cordycepin	50, 100, and 200 g per kg / LPS-induced acute lung injury mice model	In vivo/ Male BALB/c mice	Inhibition of Nrf2 and HO-1 expressions, MDA content, IL-1β, TNF-α and NF-κB activation.	[133]
*C. militaris* and *Rumex crispus* Mixture	50 and 100 µg per mL/ LPS-induced splenocytes	Ex vivo/ splenocytes	Suppressed COX-2, iNOS, IL-1β, IL-6, TNF-α, IFN-γ) and NO synthesis	[135]
*C. militaris*-based nanoemulsion	25 and 50 µg per mL/ LPS-induced Macrophages	In vitro/ RAW264.7 Macrophages	Reduced expression of proinflammatory cytokines (TNF-α, IL-1β, IKKa, iNOS, IL-6, NF-kß) and NO production.	[171]
Mulberry leaves fermented with *C. militaris*	100, 200 and 400 μg per mL/ LPS-induced Macrophages	In vitro/ RAW264.7 Macrophages	Anti-inflammatory activity by iNOS-mediated COX-2, expression of inflammatory cytokines (IL-1β, IL-6 and TNF-α), and MAPK signaling pathway	[148]
Cordycepin	10, 50 and 100 μM/ IL-1β-stimulated human osteoarthritic chondrocytes	Ex vivo/ osteoarthritic chondrocytes	Suppressed IL-1β, PGE2, MMP-13, IL-6, iNOS, COX-2 and NO synthesis.	[145]
Cordycepin	PBMCs (Kawasaki disease patients), LPS-induced Macrophages	In vitro and Ex-Vivo/ PBMCs, macrophages	Inhibition of LPS-stimulated TNFα production in mouse macrophages and in PBMCs	[172]
Cordycepin	1, 5, 10 and 20 mg per kg/ Traumatic brain injury	In vivo/ Sprague-Dawley rats	Increased arginase 1 and IL-10. Inhibition of IL-1β, iNOS, MPO and MMP-9, and NADPH oxidase expression.	[173]
*C. militaris* fruiting bodies extract	4 g per kg/ OVA sensitized airway inflammatory mice model	In vivo/ BALB/c mice	Inhibited asthmatic airway inflammation and blocked bronchoconstriction mediators-leukotrienes	[97]
*C. militaris*, *C. militaris* fermented *Haliotis discus hannai* (HFCM-5)	50, 100 and 200 µg per mL/ LPS-induced Macrophages	In vitro/ RAW264.7 Macrophages	Decreased proinflammatory cytokines, TNF-α and IL-6 in a concentration-dependent manner. In addition, showed nitric oxide inhibitory activity.	[174]
Cordycepin	50 and 100 μM/ Palmitic acid and oleic acid in inflammation in Hepatocytes	In vitro/ hepatocytes	Attenuated the increased expression of inflammatory genes (TNF-α, IL-1β, Cxcl10, Ccl2 and Ccl5)	[121]
Cordycepin	100 and 200 mg per kg/ Lipotoxic model), nonalcoholic steatohepatitis	In vivo/ Mice	Suppressed inflammatory genes (IL-1β, Cxcl2, Cxcl10, Ccl2, and Ccl5), activation of NF-κB signaling, and inflammatory cell infiltration. Anti-inflammatory effects through AMPK pathways	[121]
Spent mushroom (*C. militaris*)	0.5, 1 and 1.5 g per kg/	In vitro/ pigs	Improved health conditions. Inhibition of IL-1β and TNF-α.	[175]
Fermented *cultured C. militaris* (GRC-SC11)	0–300 µg per mL/ allergic model (RBL-2H3 cells)	In vitro/ RBL-2H3	IL-4 and TNF-α inhibition	[176]
Cordycepin	2 g per liter in drinking water/ LPS stimulated animals	In vivo/ Male broilers (Ross 308)	Inhibition of COX-2 and iNOS	[123]
*C. militaris* powder	3, 1.5, and 0.5 g powder per individual/	In vivo/ Humans	Suppressed inflammatory cytokines including EGF, eotaxin, fractalkine, IP-10, IL-1α, -6, -8, IFN-α2, -γ, MIP-1α, -1β, GRO, G-CSF, GM-CSF, MCP-1, sCD40L, TGF-α, VEGF	[137]

Abbreviations: 1-fluoro-2,4-dinitrofluorobenzene: DNFB; Adipose-derived mesenchymal stem cells: ADMSCs; *Cordyceps militaris* grown on germinated *Rhynchosia nulubilis*: GRC; Cyclooxygenase 2: COX-2; Dextran sodium sulfate-induced: DSS; Epidermal growth factor: EGF; Fibroblast growth factor-2: FGF-2; Granulocyte-colony stimulating factor: G-CSF; Granulocyte-macrophage colony-stimulating factor: GM-CSF; GRC: fermented with *Pediococcus pentosaceus* ON89A isolated from onion hexane extract: GRC-ON89A; Growth regulated oncogene: GRO; Interferon-α2 (IFN-α2), IFN-γ; Heme oxygenase-1: HO-1; IFN-γ inducible protein 10: IP-10; Inducible NO synthase: iNOS; Intercellular adhesion molecule 1: ICAM-1; Interleukin-1α: (IL)-1α, IL-1β, IL-1ra, IL-2, IL-3, IL-4, IL-5, IL-6, IL-7, IL-8, IL-9, IL-10, IL-12(p40), IL-12(p70), IL-13, IL-15, IL-17; Lipopolysaccharide: LPS; Macrophage inflammatory protein-1α: (MIP)-1α, MIP-1β; Macrophage-derived chemokine: MDC; Malondialdehvde: MDA; Monocyte chemoattractant protein-1: (MCP)-1, MCP-3; Monocyte chemoattractant protein-1: MCP-1; Nuclear factor erythroid 2–related factor 2: Nrf2; Nuclear factor-κB: NF-κB; Mitogen -activated protein kinases: MAPKs; Ovalbumin: OVA; Peripheral Blood Mononuclear Cells: PBMCs; Transforming growth factor-α: TGF-α; Tumor necrosis factor alpha: TNF-α; Tumor necrosis factor-α: (TNF)-α, TNF-β; Vascular endothelial growth factor: VEGF; Vascular adhesion molecule 1: VCAM-1.

## 5. Functional Resemblance of *Cordyceps militaris* and Nonsteroidal Inflammation Preventing Drugs

The anti-inflammatory potential of *C. militaris* shows it has similar molecular targets to those of steroidal and nonsteroidal drugs. Nonsteroidal inflammation preventing drugs and *C. militaris* both reduce inflammation related mechanistic cascades including

➢ Production of ROS
➢ Triggering and augmented production of pro-inflammatory cytokines
➢ Inflammation-associated markers, pro-inflammatory cytokine mediated regulation of CAMs
➢ NF-κB activation
➢ Enhancing the production of arachidonic acid metabolites

Various contributing factors, such as autoimmune injury, infections, trauma, toxins, postischemia, and microbes elicit inflammatory events, Usually these responses are a part of healing or eradicating the infectious agent. Prolonged or chronic inflammation can cause persistent tissue damage through collagen and leukocytes. Inflammation for prolonged periods leads to the chronic disorders, while inhibition or downregulation of the above-indicated inflammatory responses can avert disease development or lessen its severity. Therefore, *C. militaris* may be noteworthy as an anti-inflammatory substance due to its effects on NF-κB and other inflammation-linked events for treating inflammation and chronic ailments. Furthermore, improved knowledge of bioavailability and proper consumption, such as daily recommended intake, would improve its efficacy as a therapeutic food for inflammation control.

## 6. Limitations and Future Prospects

Naturally occurring *C. militaris* is exceptionally expensive because of its rarity, host specificity, and habitat. Furthermore, due to practical constraints, such as rigorous growing conditions, cordycepin production from this medicinal fungus is unlikely to reach commercial levels. It is important to highlight that the production of cordycepin through chemical procedures is a time-consuming procedure that results in decreased productivity, as well as the discharge of a considerable volume of organic solvents that are disadvantageous for our environment. Moreover, natural production of significantly important constituents from *C. militaris* such as cordycepin on a commercial level is carried out by culturing this fungus. Although cultivation on a large scale has some concerns, several studies have reported the similarity in quantity and the nature of chemical constituents in cultivated and natural *C. militaris.*

*C. militaris* as a therapeutic agent has a number of limitations including negligence, lack of suitable systematic information, inadequate in-depth research and awareness. Formerly, it was solely used by an elite class of people but with the passage of time has become available universally. Research on *C. militaris* in terms of its therapeutic effectiveness, adverse effects, biosafety, biosecurity, and appropriate standardization should be prioritized not only by individual producers, but also by different industries including functional foods, pharmaceutical, and others. 

The raw sources for natural medicines come from nature. The majority of medications derive from botanical origins. People select these medications for a variety of reasons, including the fact that chemically created pharmaceuticals may cause patients to become sicker, whereas natural measures such as medicinal mushrooms may combat life-threatening diseases with lower side effects on human health. The pharmaceutical industry requires innovation providing low-cost materials with appropriate health security in order to maintain healthy growth. The combination of chemistry and biotechnology with bio-originated starting materials, such as secondary metabolites, has the potential to revolutionize edible mushroom-based pharmaceuticals. As a result, *C. militaris* derived metabolites, including cordycepin, peptides, polysaccharides, and other active constituents, will be a major driving force in the field of green pharmacognosy and pharmacology. 

## 7. Concluding Remarks 

Medicinal fungi are classical examples of natural riches pharmacological importance. Our review encompasses the therapeutic effects of *C. militaris* in the context of ameliorating inflammation-related conditions and disorders. This species has gained importance as a potent bio-functional food source due to its diverse biological activities against inflammation-mediated disorders such as diabetes, allergies, obesity, infectious diseases and cancer. Different bio-functional components from the species are able to regulate inflammation at the molecular level. The inhibition of inflammation at the molecular level proceeds through various mechanisms including inhibition of prostaglandins, cytokines and chemokines, MMPs, oxidative stress, and suppressed inflammation associated transcription factors. In the pharmaceutical sector, *C. militaris*-associated bioactive ingredients may result in the development of a viable base for pharmaceutical industries for treatment and management of emerging diseases. In this regard, the species needs to be investigated by contemporary scientific methods including gene sequencing, precise analysis of bioactive chemicals and their screening, as well as pharmacological research and clinical trials. The food industry is already generating formulations with *C. militaris* constituents but two important parameters that require extensive work are the development of a complete safety profile, bioavailability studies and standardization of the pharmacodynamics parameters, as well as consideration of regulatory aspects. 

## Data Availability

Not applicable.
